# American Medical Association—The Preliminary Announcement of the Committee of Arrangements

**Published:** 1898-04

**Authors:** J. W. Graham, W. A. Jayne

**Affiliations:** Chairman; 306 McPhee Building; Denver, Colo.; Secretary; 306 McPhee Building; Denver, Colo.


### American Medical Association—The Preliminary Announcement of the Committee of Arrangements. Denver Meeting, June 7-10, 1898.

306 McPhee Building.
Denver, Colo., February 23, 1898.

The Committee of Arrangements announces that preparations for the coming meeting are well advanced. A large number of prominent men have signified their intention to be present and read papers, and an excellent scientific program is assured. The indications all point to a large and successful meeting.

Convenient and ample accommodations have been secured for the general sessions, section work, registration, and exhibits.

The entertainment of members and their families is being planned on an elaborate scale, and the committee promises all who may come a most enjoyable time.

Denver is an interesting city, and the State offers many and varied attractions to visitors.

Local excursions are being arranged to take place after the meeting, that all may have ample opportunity of visiting various points of interest in the State and seeing the best scenery of the Rocky Mountains.

The committee confidently expects to obtain a one-half rate and thirty-day limit for the round trip on roads west of Chicago and St. Louis, and reduced rates on eastern roads. The rates will be announced in the journal of the Association as soon as definitely determined.

The following is a list of the principal hotels of Denver with the rates agreed upon for the meeting:

The Brown Palace Hotel, 17th street and Broadway. Take 17th street (red) car to hotel. European plan, $1.50 per day and upward. American plan, $3.00 to $5.00 per day and upward. These regular rates will apply during the meeting for available rooms if no reservation has been made. For reservations and rooms to be occupied by one person only, application must be made to hotel for rates.

The Windsor Hotel, 18th and Larimer streets. Take 17th street car to Larimer street. American plan, $2.00 per day; room with bath, $2.50 per day.

The Albany, 17th and Stout streets. Take 17th street (red) car to hotel. American plan, $2.00 to $3.50 per day.

The Markham Hotel, 17th and Lawrence streets. Take 17th street car to hotel. European plan, $1.00 per day and upward.

All reservations at the Albany and Markham must be for the full term of the meeting, and be paid for whether occupied or not unless cancelled ten days in advance. They reserve right to place persons occupying a room alone in a single room.

The New St. James Hotel, Curtis near 16th street. Take 17th street (blue) car to hotel. American plan, $2.00 to $3.50 per day.

L'Imperiale, 14th street and Court Place. Take Colfax Avenue car to Court Place. American plan, $2.00 to $3.00 per day.

Metropole Hotel, Broadway near 17th street. Take 17th street (red) car to Broadway. European plan, $1.00 to $2.00 per day. $1.00 rooms, $1.50 if occupied by two persons. No reservations made.

The Oxford Hotel, 17th and Wazee streets. Two blocks above depot. European plan, $1.00 to $3.00 per day. An extra charge for two persons in one room,

The American House, 16th and Blake streets. Take 16th street car to Blake street. American plan, $2.00 per day.

The above hotels make no extra charge for reservations or for persons occupying a room alone except as stated.

The Hotel Broadway, Cheyenne street and Broadway. Take Colfax Avenue car to Cheyenne street. American plan, $1.25 to $1.50 per day.

The Vallejo, 1420 Logan Avenue. Take Colfax Avenue car to Logan Avenue. American plan, $2.00 per day and upward.

The Devonshire, 14th and Logan Avenues. Take Colfax Avenue car to Logan Avenue. American plan, $1.50 per day and upward.

The Albert, 17th and Welton streets. Take 17th street (red) car to hotel. European plan, $1.00 to $1.50 per day.

The Aldine, 1013 Seventeenth Avenue. Take 17th street (red) car to hotel. American plan, $1.25 per day.

The Richelieu, 1727 Tremont street. Take 17th street (red) car to Tremont street. European plan, 50 cents to $1.00 per day.

The Earl, 1430 Tremont street. Take Colfax Avenue car to Tremont street. American plan, $1.50 to $2.00 per day.

Glenarm Hotel, Glenarm and 15th streets. Take Colfax

Avenue car to hotel. European plan, $1.00 per day and upward.

The Bonaventure, 18th and Glenarm streets. Take 17th street (red) car to Glenarm street. European plan, 50 cents to $1.50 per day.

The Drexel, 17th and Glenarm streets. Take 17th street (red) car to hotel. European plan, 75 cents to $1.50 per day.

These hotels make small extra charges for two persons occupying same room. No charge for reservation.

Applications for rooms should be made to the hotels direct. For special information apply to Robert Levy, M. D., Chairman Sub-Committee on Hotels, California Building, Denver, Colo.

J. W. GRAHAM, M. D., Chairman.

W. A. JAYNE, M. D., Secretary.

